# Supplementary material for: Spectral signatures of a unique charge density wave in Ta2NiSe7
Source: Nat Commun. 2023 Jun 9;14:3388. doi: 10.1038/s41467-023-39114-z (PMC10256806; doi:10.1038/s41467-023-39114-z)
Supplement: Supplementary file 1 — Supplementary Information [file 41467_2023_39114_MOESM1_ESM.pdf]

Supplementary Information:

Spectral signatures of a unique charge density wave in Ta_2NiSe_7

Matthew D. Watson,[1,*] Alex Louat,[1] Cephise Cacho,[1] Sungkyun Choi,[2,3] Young Hee Lee,[2,4] Michael Neumann,[2,3] and Gideok Kim[2,3]

[1]*Diamond Light Source Ltd, Harwell Science and Innovation Campus, Didcot, OX11 0DE, UK*

[2]*Center for Integrated Nanostructure Physics (CINAP), Institute for Basic Science (IBS), Suwon 16419, Republic of Korea*

[3]*Sungkyunkwan University, Suwon 16419, Republic of Korea*

[4]*Department of Energy Science, Sungkyunkwan University, Suwon 16419, Republic of Korea*

(Dated: May 18, 2023)

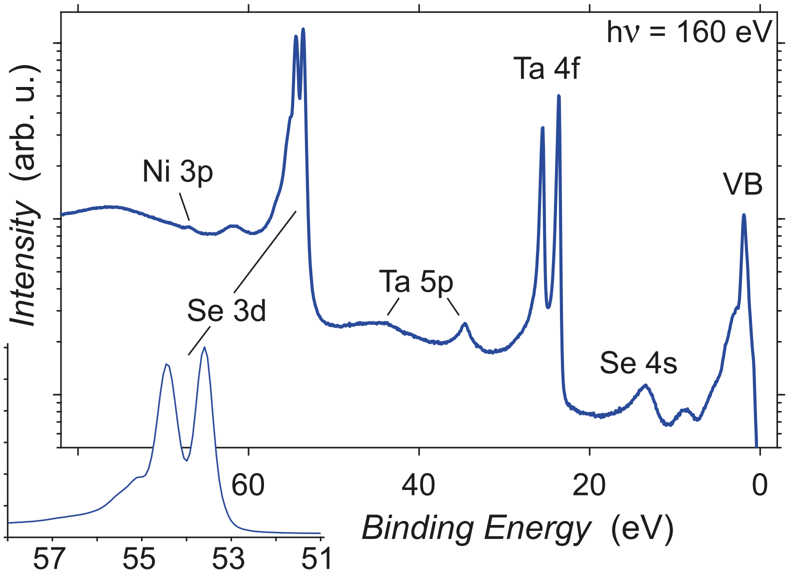

Supplementary Fig. 1. **Se 3d core levels.** The feature-rich Se $3d$ shallow core levels (inset 1) are indicative of the varying valence charges on each of the 7 distinct Se sites in the structure. On the other hand, the Ta $4f$ states (inset 2) appear as a “clean" doublet, not showing any resolvable splitting due to the distinct chemical environments of Ta1 and Ta2. However this can be expected, partly because the more localised $4f$ wavefunctions are less sensitive to chemical shifts, but also because both of these are expected to have a formal valence of approximately $\mathrm{Ta}^{5+}(5\mathrm{d}^0)$, albeit that a slight occupancy of the Ta2 5d-derived conduction band will turn out to play an important role near E_F. We additionally find a weak and broad feature at around 13.5 eV binding energy that corresponds to the Ni $3p$.

* matthew.watson@diamond.ac.uk

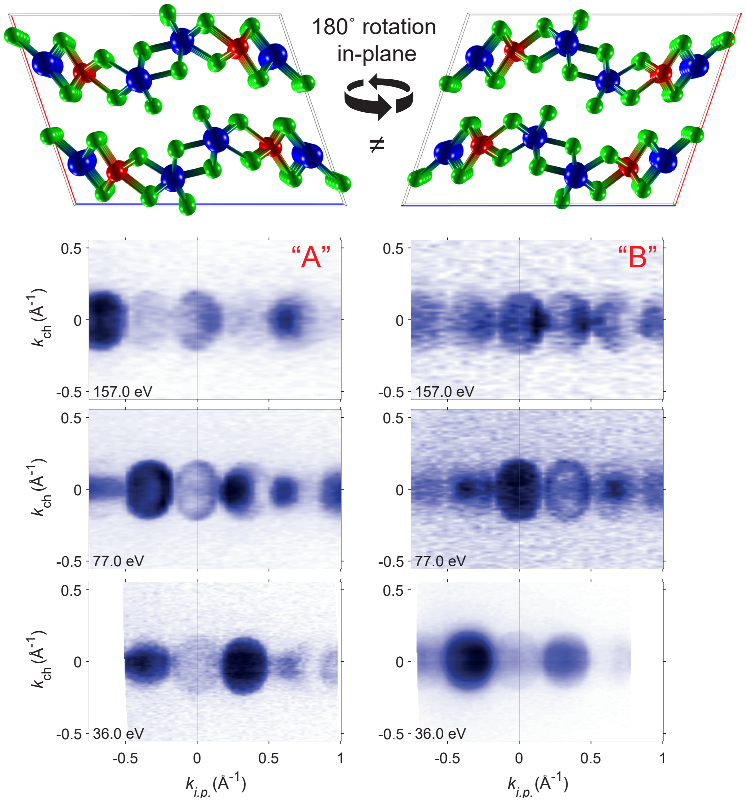

Supplementary Fig. 2. **Absence of mirror symmetry.** As mentioned in the main text, there is a mirror plane perpendicular to k_{ch}, but no mirror plane perpendicular to $k_{i.p.}$ or twofold rotation symmetry about the axis normal to the cleavage. Thus, after aligning the sample so that k_{ch} is along the analyser slit direction (vertical, at the I05 beamline), there can be two inequivalent orientations of the sample. The data presented in this figure were all obtained from the same sample, but the azimuthal orientation of the sample for the data labelled B are 180° was rotated compared to A. Although the bands are formally the same at normal emission, as soon as you move away with $k_{i.p}$ the band dispersions are inequivalent. Moreover, even at normal emission it appears that the matrix elements are somewhat different between the two cases. The data in Fig. 2 and 4 of the main text are from the A orientation, while Fig 3 is from the B orientation.

The data presented here also underlines one of the experimental challenges with measuring Ta_2NiSe_7, which is that there are so many Fermi surfaces measured in the $k_{i.p} - k_{ch}$ plane at any given photon energy, that is not trivial to correctly identify the normal emission.

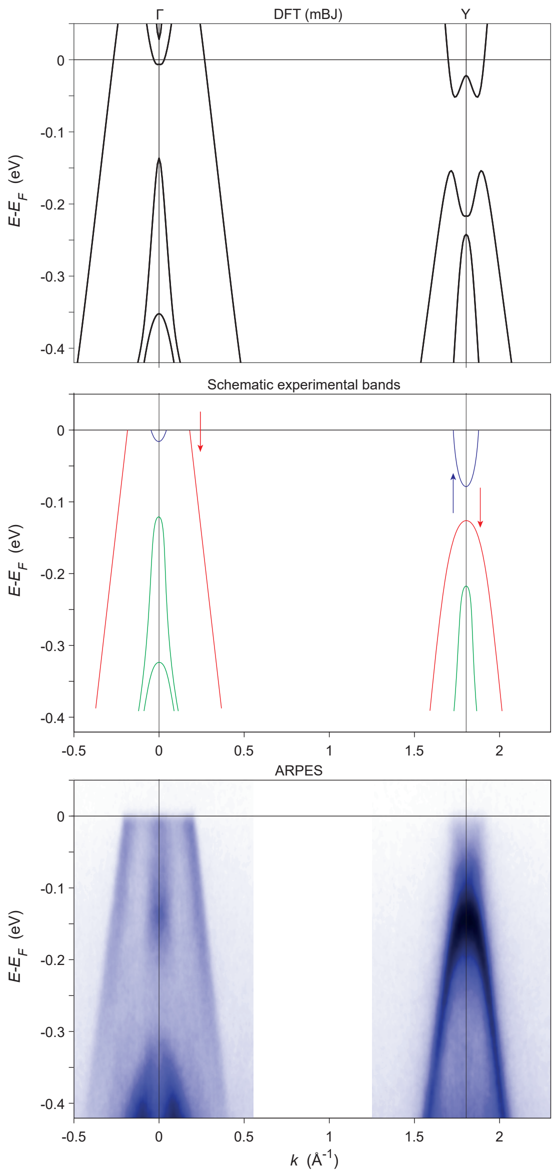

Supplementary Fig. 3. **Comparison of data with DFT.** Comparison of our DFT calculation using the mBJ potential (top), with a schematic diagram of the experimental band positions. At the Γ point, the main difference is that the uppermost hole-like band, which has Ni/Se character, is experimentally found to be shifted downwards, and the resulting Fermi surface is smaller in experiment than in theory. At the Y point, the calculation would suggest that the Ta2-derived electron-like dispersion should dip below the uppermost valence bands, and a sizeable hybridisation gap might occur. Experimentally, however, we find that the uppermost valence band at Y is deeper than predicted (shifted downwards, consistent with the equivalent band at Γ, while the electron-like dispersion is shallower than predicted at Y (shifted upwards), with the result that the states don't actually cross and no hybridisation gap is visible. Thus overall we can say that at the Fermi level the experiments are qualitatively and topologically equivalent to the calculated Fermi surface, but the sizes of the pockets are smaller, and there are some differences in the valence bands. Bottom panel shows the ARPES data (adapted from Fig. 4 in the main text.

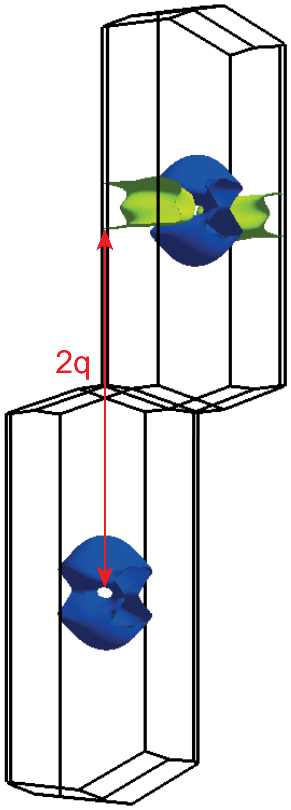

Supplementary Fig. 4. **Illustration of nesting at 2q in the DFT Fermi surface.** For clarity, the electron-like Fermi surface is not plotted in the lower BZ.

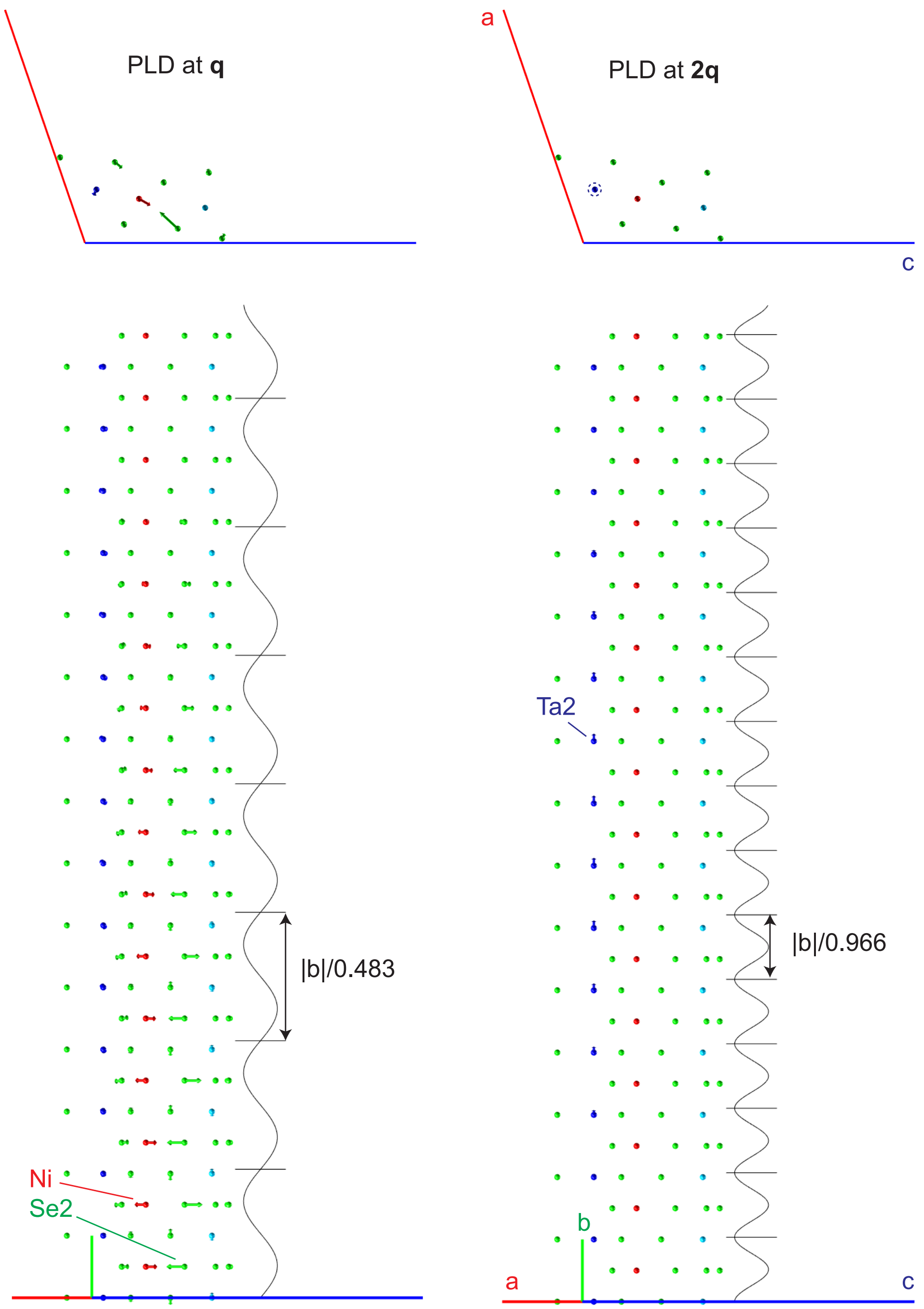

Supplementary Fig. 5. **Visualisation of the Periodic Lattice Distortion.** Full 3D representation of the periodic lattice distortion (PLD) in the CDW phase, showing the component at q and 2q separately. For simplicity only one of the Ta_2NiSe_7 formula units is shown (there are 4 in the full conventional unit cell). It can be seen that the modulation at **q** is mainly transverse, and strongest on the Ni and Se2, while the modulation at **2q** is almost entirely on the Ta2. The atomic positions and distortions are taken from Lüdecke *et al.* [1]. Arrows exaggerate distortions by a factor of 10.

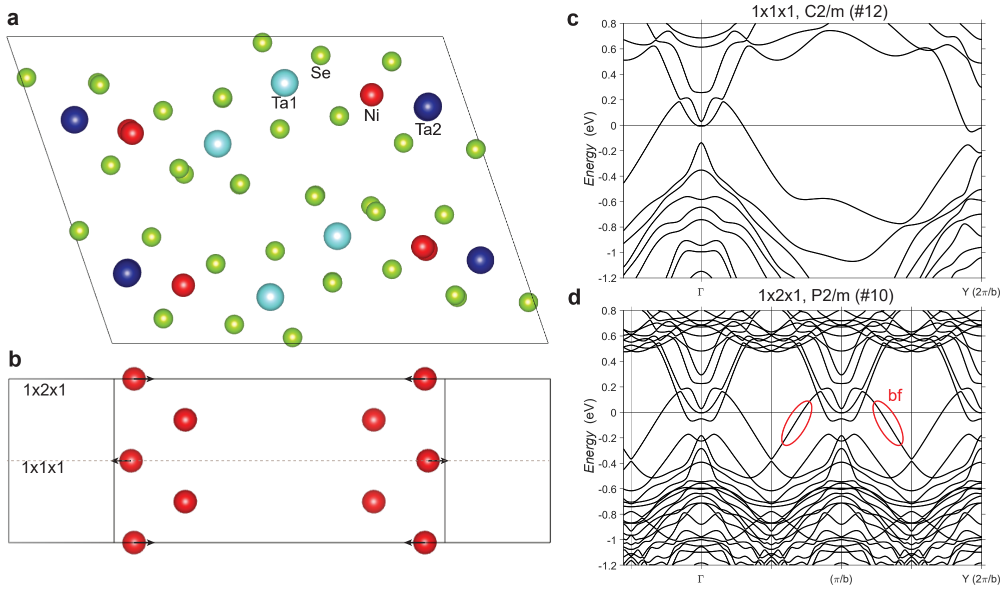

Supplementary Fig. 6. **Supercell DFT calculation.** Since the CDW periodicity is close to commensurate, we performed a $1 \times 2 \times 1$ superstructure calculation, corresponding to a commensurate q=(0,0.5,0), primarily with a view to give insights into the backfolded bands observed at low temperatures. We considered only the transverse components of the instability at q, and ignore small displacements along the b direction, i.e. all atoms remain in high-symmetry planes. The $2q$ modulation is not included. The atomic displacements are taken from Lüdecke *et al.* [1], taking care of phase factors. The displacements of atoms related by the C-centering of the undistorted unit cell (i.e. intra-unit cell translation by (0.5,0.5,0)) are $\pi/2$ out of phase. This leads to zero distortion for certain atomic chains, as shown in (b) for the Ni atoms. For the true case of incommensurate periodicity, however, the displacements in every chain would become large at some distance along the chain (see also Fig. SM5). The overall symmetry of this $1 \times 2 \times 1$ supercell was found to be $P2/m$ (space group 10), i.e. the $2/m$ symmetry is preserved. The unit cell is twice as large as the 1x1x1 conventional unit cell (four times as large as the primitive unit cell), now containing 8 formula units and 30 inequivalent atoms. We used the same mBJ potential and included spin-orbit coupling, as for the main calculations, albeit that a much smaller k-grid of $3 \times 7 \times 4$ was used for convergence due to the much increased computational workload. The calculated band dispersions in (d) can be understood as a superposition of original and folded bands, as the $1 \times 2 \times 1$ supercell maps the bands from the Y point to Γ. Furthermore the new periodicity introduces eigenstate around (π/b), highlighted in (d), resembling the bands which are backfolded at $q = 0.483 * (2\pi/b)$ in the experiments. However these calculations show no clear evidence for any sizeable hybridsation effects, and further work is needed to understand the full energetics of the problem.

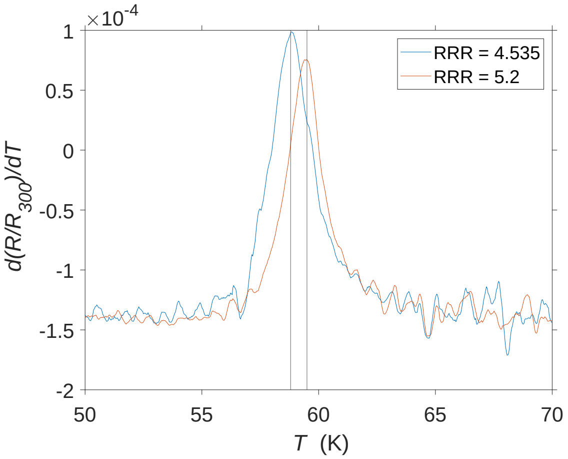

Supplementary Fig. 7. **Determination of Tc from resistivity.** Plot shows the derivative of (normalised) resistivity with respect to temperature. The red curve corresponds to the RRR = 5.2 sample, for which the resistivity data is shown in Fig. 1b. of the main text. The equivalent plot for a slightly lower RRR sample from the same batch is also shown. The peak in dR/dT is used to determine T_c values of 59.5 and 58.8 K. These two data points (RRR=5.2, Tc=59. K; RRR=4.5, Tc=58.8 K) align well with the trend found from other samples in the literature, as seen in Fig. 1(c) of He *et al.* [2].

Supplementary Table I. **Single crystal diffraction.** To confirm that our samples are comparable to those in the literature, X-ray diffraction (XRD) was performed using a four-circle diffractometer (Rigaku, XtaLAB PRO) with Cu K_α radiation on a Ta_2NiSe_7 single crystal. Detected peaks were indexed by a monoclinic domain and were integrated for refinements. An absorption correction was done using a multifaceted crystal model via the empirical absorption correction. The JANA2006 software was utilized for structural refinements and a Shelx extinction model implemented in the software was chosen. The data were collected at room temperature. Table I presents structural parameters of Ta_2NiSe_7 obtained from single-crystal XRD refinement. The space group is $C2/m$ (No. 12) (a=13.8508(18)Å, b=3.4812(4)Å, c=18.6003(12)Å, β=109.000(10)°, Z=4). Agreement factors are R(obs)=4.53%, wR(obs)=4.51%, GOF(obs)=1.64. 257 independent peaks were used after the average of selective peaks. The criteria for selecting peaks is $I/\sigma(I) > 3$, where I is the averaged intensity of equivalent peaks and σ is an estimated standard deviation. All sites are assumed to be fully occupied. U is the isotropic thermal parameter. The refined parameters are in good agreement with the structure reported in the previous literature [3].

Atom	Site	x	y	z	$U(^2)$
Ta1	4i	0.1509(1)	0	0.3992(1)	0.0063(4)
Ta2	4i	0.2288(1)	0	0.0898(1)	0.0060(4)
Ni1	4i	0.6869(3)	0	0.2079(2)	0.0067(8)
Se1	4i	0.3613(2)	0	0.0122(1)	0.0060(5)
Se2	4i	0.5630(2)	0	0.2952(1)	0.0082(5)
Se3	4i	0.5830(2)	0.5	0.1377(1)	0.0055(5)
Se4	4i	0.8484(2)	0	0.1744(1)	0.0096(6)
Se5	4i	0.0189(2)	0.5	0.4178(1)	0.0066(6)
Se6	4i	0.1989(2)	0	-0.4468(1)	0.0087(6)
Se7	4i	0.2589(2)	0	0.2997(1)	0.0076(6)

[1] Lüdecke, J., Schneider, M. & van Smaalen, S. Independent q and 2q Distortions in the Incommensurately Modulated Low-Temperature Structure of $NiTa_2Se_7$. *Journal of Solid State Chemistry* **153**, 152–157 (2000).

[2] He, J. *et al.* Band dependence of charge density wave in quasi-one-dimensional Ta_2NiSe_7 probed by orbital magnetoresistance. *Appl. Phys. Lett.* **111**, 052405 (2017).

[3] Sunshine, S. A. & Ibers, J. A. Synthesis, structure, and transport properties of tantalum nickel selenide (Ta_2NiSe_7) and tantalum platinum selenide (Ta_2PtSe_7). *Inorg. Chem.* **25**, 4355–4358 (1986).
